# Impaired insulin/IGF-1 is responsible for diabetic gastroparesis by damaging myenteric cholinergic neurones and interstitial cells of Cajal

**DOI:** 10.1042/BSR20170776

**Published:** 2017-10-27

**Authors:** Shu Yang, Bo Wu, Haimei Sun, Tingyi Sun, Kai Han, Dandan Li, Fengqing Ji, Guoquan Zhang, Deshan Zhou

**Affiliations:** 1 Department of Histology and Embryology, School of Basic Medical Sciences, Capital Medical University, Beijing 100069, P.R. China; 2 Beijing Key Laboratory of Cancer Invasion and Metastasis Research, Beijing 100069, P.R. China; 3 Department of Histology and Embryology, Logistics University of Chinese People’s Armed Police Force, Tianjin 300162, P.R. China

**Keywords:** Diabetic gastroparesis, IGF-1, Insulin, Interstitial cell of Cajal, Myenteric cholinergic neuron, Stem cell factor

## Abstract

Diabetic gastroparesis is a common complication of diabetes mellitus (DM) that is characterized by decreased serum insulin and insulin-like growth factor-1 (IGF-1). Despite the fact that insulin treatment not glycemic control potently accelerated gastric emptying in type 1 DM patients, the role of insulin/InsR and IGF-1/IGF-1R signaling in diabetic gastroparesis remains incompletely elucidated. In the present study, type 1 DM mice were established and treated with insulin or Voglibose for 8 weeks. The gastric emptying was delayed from DM week 4 when the gastric InsR and IGF-1R were declined. Meanwhile, the gastric choline acetyltransferase (ChAT) was significantly reduced and the myenteric cholinergic neurones and their fibers were significantly diminished. The production of stem cell factor (SCF) was dramatically repressed in the gastric smooth muscles in DM week 6. TWereafter, interstitial cells of Cajal (ICC) were clearly lost and their networks were impaired in DM week 8. Significantly, compared with Voglibose, an 8-week treatment with insulin more efficiently delayed diabetic gastroparesis development by protecting the myenteric cholinergic neurones and ICC. In conclusion, diabetic gastroparesis was an aggressive process due to the successive damages of myenteric cholinergic neurones and ICC by impairing the insulin/InsR and IGF-1/IGF-1R signaling. Insulin therapy in the early stage may delay diabetic gastroparesis.

## Introduction

Diabetes mellitus (DM) is the leading dismetabolic chronic disease with a global prevalence approaching 400 million people [1]. Long-standing DM often results in systemic complications including cardiovascular diseases, neuropathy, retinopathy, nephropathy, as well as gastroenteropathy. Diabetic gastroparesis, characterized by early satiety, nausea, vomiting, postprandial fullness, abdominal pain, and delayed gastric emptying without evidence of mechanical obstruction [2], occurs in approximately 50% of type 1 DM patients and 30% of type 2 DM patients. Although the diabetic gastroparesis is not malignant, it inhibits absorption of nutrients and oral antidiabetic medicine, which could interfere with glycemic control and lead to an inefficient treatment of DM. The pathogenesis of the diabetic gastroparesis is still under research and it is believed to be multifactorial, e.g. hyperglycemia, enteric nervous system (ENS) injury, myopathy, loss of interstitial cells of Cajal (ICC) etc. [3–6]. Although there have been substantial advancement in our understanding of the underlying cellular dysfunction of the diabetic gastroparesis, how the ENS and ICC are damaged in diabetic gastroparesis remain poorly known.

DM is characterized by a deficiency (type 1 DM) or resistance (type 2 DM) to insulin, apart from which, a decrease in insulin-like growth factor-1 (IGF-1) was documented in both type 1 and type 2 diabetic patients [7,8]. Insulin and IGF-1 share structural homologies, so do their receptors, InsR and IGF-1R, respectively. Thus there is a physiological and pharmacological cross-talk between insulin/InsR and IGF-1/IGF-1R systems [9]. Evidence have shown that insulin/InsR and IGF-1/IGF-1R signaling were implicated in the development of nervous system by promoting neuronal growth, survival, proliferation, and differentiation [10,11]. Impaired insulin/InsR and IGF-1/IGF-1R signaling were closely related with diabetic peripheral neuropathy, which could be ameliorated or prevented by insulin or IGF-1 treatment [11–13]. However, much less is known about the role of insulin/InsR and IGF-1/IGF-1R signaling in the ENS and ICC during the development of the diabetic gastroparesis. A pilot trial on patients with type 1 DM revealed that insulin treatment efficiently accelerated gastric emptying [14], suggesting a potential role of insulin/InsR signaling in the diabetic gastroparesis.

Therefore, the present study investigated the alterations of the myenteric neurones and ICC within the gastric wall of type 1 DM mouse model, in order to evaluate the effect of IGF-1/IGF-1R and insulin/InsR signaling on the development of diabetic gastroparesis.

## Materials and methods

### Establishment of type 1 DM mouse model

Male BALB/c mice (6 weeks, 22–26 g) were purchased from the Animal Center of Capital Medical University (Beijing, China). All mice were maintained in a temperature-controlled room (23 ± 1°C), with a constant 12 h-light/dark cycle. Food and water were available *ad libitum*. Each mouse received a single intraperitoneal injection of Alloxan monohydrate (200 mg/kg, Sigma–Aldrich) [15,16]. The littermates receiving the same dose of normal saline (NS) were set as controls. Fasting intravenous blood glucose was measured by an Accu-Chek Active Complete blood glucose monitor (Roche) 72 h later. The mice with blood glucose ≥11.1 mmol/l were considered as type 1 DM mice and used in the present study. In total, 135 DM mice were successfully established and blood glucose was monitored weekly. In DM week 2, 4, 6, and 8, mice were killed by an overdose of 4% chloral hydrate (0.02 ml/g). A separate group of 60 DM mice daily received an intraperitoneal injection of insulin (0.04 U/g, Wanbang Biopharmaceuticals, China) or oral antidiabetic drug of Voglibose (0.5 mg/mouse, Cisen Biopharmaceuticals, China) for 8 weeks. In addition, 15 DM mice daily received no).The subdivision of the total mice is indicated in Supplementary Table S1. All experimental procedures were in accordance with the National Institutes of Health guide for the care and use of Laboratory animals (NIH Publication number 8023, revised 1978) and approved by the Institutional Animal Care Committee from Capital Medical University, Beijing, China.

### ELISA

At the end of 2, 4, 6, and 8 weeks, 1.5 ml of blood was collected from eyes of each mouse anesthetized by 4% chloral hydrate (0.01 ml/g). Serum insulin and IGF-1 were measured with corresponding ELISA Kit (Raybio technology) according to the manufacturers’ instructions. The absorbance at 490 nm was read using a microplate reader (Multiskan FC, Thermo Scientific). All experiments were repeated six times.

### Gastric emptying

Gastric emptying was evaluated according to Song and Chen [17]. Briefly, each mouse was intragastrically administered with 0.2 ml of methylcellulose-Phenol Red solution. Thirty minutes later, the mouse was anesthetized by 4% chloral hydrate (0.01 ml/g) and the stomach was removed. The gastric content was placed in 100 ml of 0.1 N NaOH and settled for 1 h at 25°C. Afterward, 5 ml of supernatant was taken from the solution and put into 0.5 ml of 20% trichloroacetic acid, then centrifuged at 3000 rpm for 30 min. The contents were then mixed with 4 ml of 0.5 N NaOH. The absorbance was determined at a wavelength of 560 nm with a microplate reader (Multiskan FC). Gastric emptying (%) = (Phenol Red absorbance – residual Phenol Red absorbance)/phenol Red absorbance × 100%.

### Isometric tension recording

Gastric content was flushed out in precooled Kreb’s solution. Smooth muscle strip (2 mm × 10 mm) was prepared and suspended vertically in an eight-channel organ bath filled with Kreb’s solution and oxygenated with 95% O_2_ and 5% CO_2_ at 37°C. One pole of the strip was fixed to an organ holder and the other pole was connected to an isometric tonotransducer. Mechanical activity of the smooth muscle strip was recorded with a computer-aided data acquisition system (Power lab biology signal recording system, AD Instruments). The initial tension was set as 1 g. The amplitude and frequency of the spontaneous contraction of the smooth muscle strip were analyzed by chart software (AD Instruments).

### Western blotting

The gastric mucosa and submucosa were removed and the smooth muscles were homogenized in cold lysis buffer (Applygen, China) and the supernatants were collected. After SDS/PAGE (10% gel), the proteins were transferred on to PVDF membranes and blocked with 5% non-fat dry milk for 1 h at 25°C. The membranes were incubated with primary rabbit anti-IGF-1R, goat anti-choline acetyltransferase (ChAT )mouse anti-SCF, or rat anti-KIT at 4°C overnight, followed by incubation with corresponding HRP-conjugated secondary antibody for 1 h at 25°C. The proteins were detected using ECL chemiluminescence (Thermo Scientific) and viewed in Fusion FX Vilber Lourmat (France). A goat anti-GAPDH was used as an internal control in all the cases. The detailed antibodies are listed in Supplementary Table S2.

### Immunofluorescence staining

To obtain whole mount preparations, mouse stomach was inflated with 4% paraformaldehyde or 100% acetone for 2 h and immersed in the same fixative for 12 h at 4°C. One sample of 0.5 cm × 0.5 cm was randomly cut from the gastric corpus and antrum. The mucosa and submucosa were removed and the longitudinal smooth muscle layer containing myenteric plexus was dissected. For cryosections, the stomach was opened along the gastric greater curvature and embedded in optimal cutting temperature compound. Cryosections (8-μm thick) were cut with a cryostat (Leica, CM3050S) and fixed with 4% PFA or 100% acetone for 30 min at 4°C.

The specimens were permeabilized with 0.3% Triton X-100 for 20 min. Non-specific binding sites were blocked with 1% BSA for 30 min. The specimens were incubated with primary rabbit anti-IGF-1R, rabbit anti-InsR, goat anti-ChAT, rabbit anti-S100, mouse anti-stem cell factor (SCF), or rat anti-KIT overnight at 4°C, followed by incubation with corresponding secondary antibody for 1 h at 25°C. The specimens were mounted with fluorescent mounting medium containing DAPI (Zhongshan Jinqiao Biotechnology, China) and visualized by a fluorescence microscope (Nikon, Ni) or confocal laser scanning microscope (Leica, TCS SP5). Specificity was verified by omitting the primary antibody and by preabsorption with appropriate blocking peptide. The detailed antibodies are listed in Supplementary Table S2.

### Image analysis

Fifteen photographs were randomly taken per whole mount preparation stained by immunofluorescence based on stereological rules at magnification of 20×. The target area of interest (AOI) was outlined and measured by Image Pro-Plus Software 6.0 (Media Cybernetics, Silver Spring). To obtain the area density of the AOI, the total area of the AOI was divided by the total area of the fields of view. To obtain the cell number density in the AOI, the total number of the cells in the AOI was divided by the total area of the fields of view.

### Statistics

Statistical analyses were performed with the SPSS 17.0 software. Data were expressed as the mean ± S.E.M. and compared using one-way ANOVA and LSD post hoc analysis. A 2*P*-value of 0.05 was adopted.

## Results

### Establishment of type 1 DM mouse model

In total, 155 mice were treated with Alloxan monohydrate. However, 20 mice died during the induction of DM by Alloxan monohydrate treatment. Finally, 135 mice had profound hyperglycemia (Supplementary Table S3), indicating the type 1 DM mouse model was successfully established. The body weight of the DM mice was less than that of the controls (Supplementary Table S3), accompanied with several manifestations, such as polydipsia, polyphagia, polyuria, and smell of ketone bodies in urine.

### Diabetic gastroparesis occurred in DM week 4

**Figure 1:**
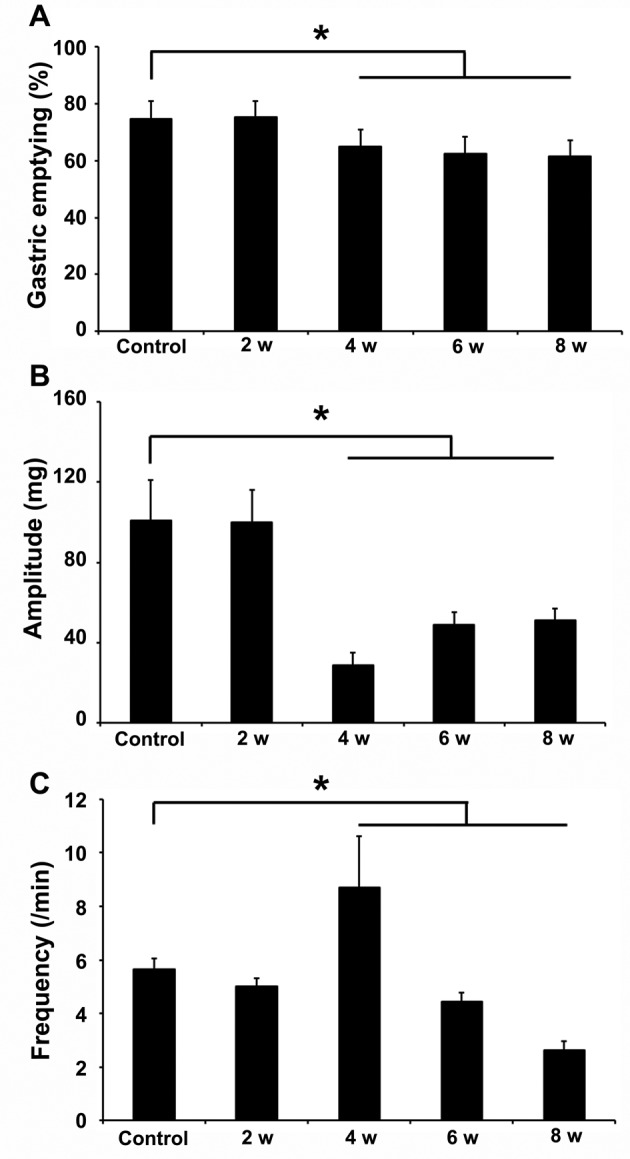
Gastric emptying was measured by the absorbance of Phenol Red solution before and after intragastric administration (*n*=5) Amplitude and frequency of spontaneous contraction were recorded by isometric tension recording (*n*=5). (**A**) The gastric emptying was significantly delayed from DM week 4 compared with the controls (*n*=5; *P<0.05). (**B**) The amplitude of spontaneous contraction was significantly reduced in DM week 4 compared with the controls. Though the amplitude was gradually increased in DM week 6, it could not completely regain the controls’ level (*n*=5; *P<0.05). (**C**) Despite the frequency of spontaneous contraction was overtly increased in DM week 4, it was visibly decreased in the DM mice afterward compared with the controls (*n*=5; *P<0.05).

To identify diabetic gastroparesis, the gastric emptying was measured at the end of DM week 2, 4, 6, and 8. In DM week 2, the gastric emptying did not differ from that of the age-matched controls, while it was significantly delayed from DM week 4 (*P<0.05, Figure 1A). The stomachs of the DM mice were evidently dilated owing to food retention compared with control mice (Supplementary Figure S1). The delayed gastric emptying and food retention denoted the occurrence of diabetic gastroparesis. The gastric emptying comes from spontaneous and rhythmic contractions of gastric smooth muscles, so we recorded the contractions of gastric muscles by isometric tension recording. In DM week 4, the amplitude of spontaneous contraction was significantly reduced while the frequency was significantly increased compared with the controls (*P<0.05; Figure 1B,C, Supplementary Figure S2). Afterward, the amplitude and frequency were both markedly decreased in the DM mice (*P<0.05; Figure 1B,C, Supplementary Figure S2).

### Gastric InsR and IGF-1R were decreased from DM week 4

**Figure 2:**
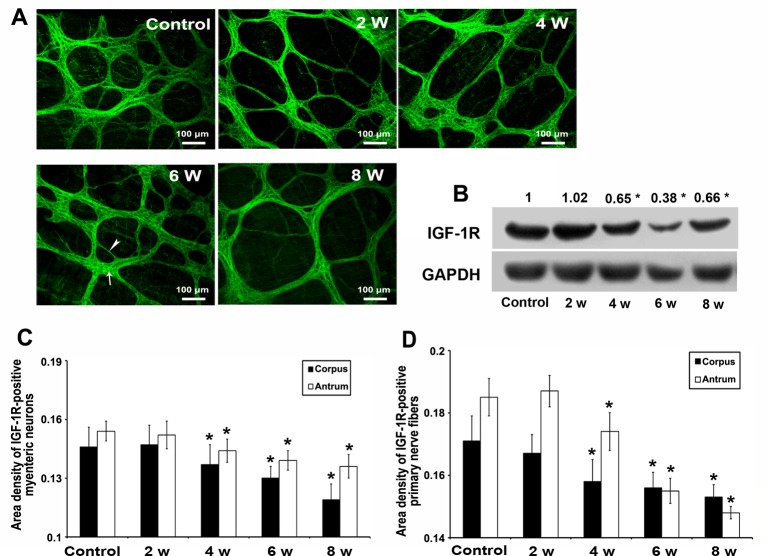
Decreased gastric IGF-1R expression and damaged neuronal networks from DM week 4. (**A**) IGF-1R^+^ myenteric plexuses including ganglion neurones (arrow) and nerve fibers (arrow head) were well exhibited via immunofluorescence staining. Neuronal networks were damaged from DM week 4 (*n*=5). (**B**) The protein level of IGF-1R in gastric smooth muscles was significantly decreased from DM week 4 compared with the controls (*n*=5; *P<0.05). (**C**) The area density of IGF-1R^+^ myenteric neurones in gastric corpus and antrum of DM mice were decreased from DM week 4 compared with the controls (*n*=5; *P<0.05). (**D**) The area density of IGF-1R^+^ primary nerve fibers in gastric corpus and antrum of DM mice were also diminished from DM week 4 compared with the controls (*n*=5; *P<0.05).

Compared with the controls, the serum insulin and IGF-1 were significantly decreased from DM week 2 (*P<0.05; Supplementary Table S4). Immunofluorescence staining showed that IGF-1R and InsR were coexpressed in the muscularis mucosa, tunica muscularis, and myenteric plexus (Supplementary Figure S3). Considering the key role of the ENS in the gastrointestinal motility, we paid special attention to the IGF-1R^+^/InsR^+^ myenteric neurones and nerve fibers in the ganglia. In DM week 2, there was no obvious change in the IGF-1R^+^ myenteric plexus or the IGF-1R protein level compared with the controls (Figure 2A,B). The area densities of the IGF-1R^+^ myenteric neurones and primary nerve fibers in the gastric corpus and antrum significantly shrunk from DM week 4 compared with controls (*P<0.05, Figure 2C,D), when the protein level of IGF-1R in the gastric smooth muscles was significantly reduced (*P<0.05, Figure 2B).

### ChAT^+^/IGF-1R^+^/InsR^+^ neurones were reduced from DM week 4

**Figure 3:**
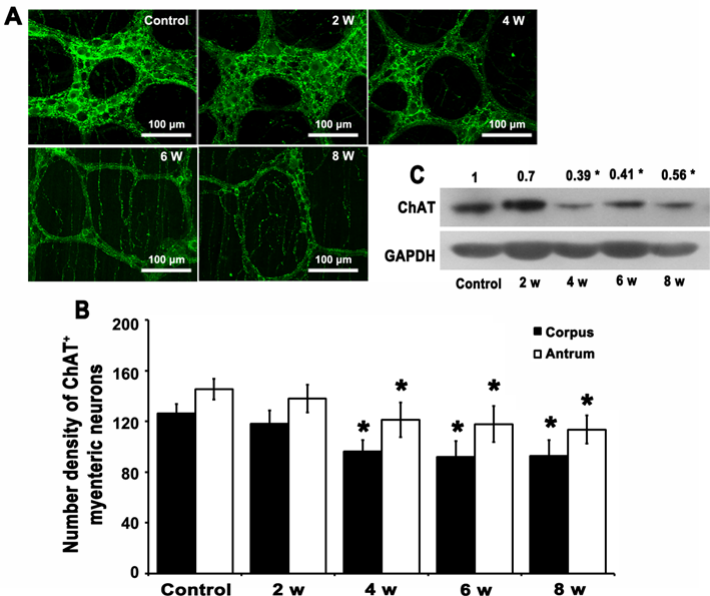
Dinimished ChAT+ neurons and gastric ChAT expression from DM week 4. (**A**) ChAT was expressed in the neurones and nerve fibers within the myenteric plexuses. ChAT^+^ cholinergic neurones were distinct, round or oval; and their processes formed neuronal networks. The immunofluorescence intensity for ChAT was gradually decreased from DM week 4 (*n*=5). (**B**) The number density of ChAT^+^ cholinergic neurones in gastric corpus and antrum of DM mice was significantly diminished from the DM week 4 compared with the controls (*n*=5; *P<0.05). (**C**) The protein level of ChAT was significantly reduced from DM week 4 compared with the controls (*n*=5; *P<0.05).

We noticed that the IGF-1R^+^/InsR^+^ cells in the myenteric ganglia were distinct, round or oval, exhibiting neurone-like features. We further confirmed that most IGF-1R^+^ or InsR^+^ cells were ChAT^+^ cholinergic neurones in the myenteric ganglia but not enteric glias by double-immunofluorescence staining, suggesting a possible role of IGF-1/IGF-1R and insulin/InsR on the ChAT^+^ cholinergic neurones (Supplementary Figures S4 and S5). In accordance with the weakened IGF-1/IGF-1R and insulin/InsR signaling, the ChAT^+^ cholinergic neurones decreased from DM week 4 (Figure 3A,B). Concomitantly, the protein level of ChAT in the stomach was significantly reduced (*P<0.05, Figure 3C). These results indicated that loss of ChAT^+^ cholinergic neurones in the gastric myenteric ganglia could be due to the impaired IGF-1/IGF-1R and insulin/InsR systems and may be responsible for the dysfunction of gastric motility.

### SCF production was lowered in DM week 6 and ICC was lost in DM week 8

**Figure 4:**
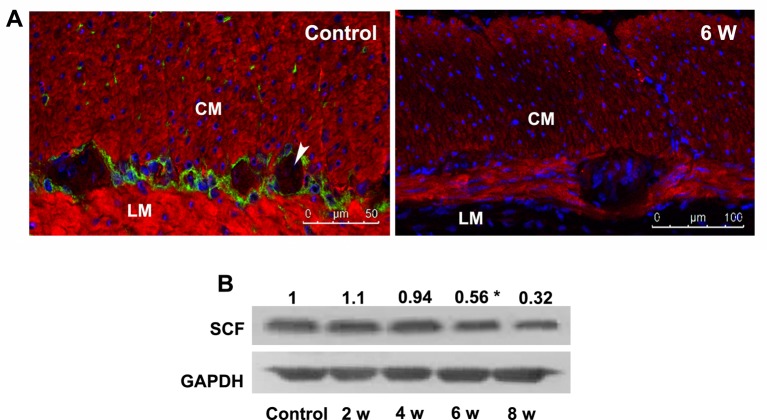
Reduced gastric SCF production from DM week 6. (**A**) SCF (red) was expressed in the smooth muscles including circular muscles (CM) and longitudinal muscle (LM) and myenteric plexuses but not in ICC (green). Immunoreactivity for SCF in the smooth muscles was much intensive than that in the myenteric plexuses (arrow head), and the immunofluorescence intensity for SCF was clearly decreased in DM week 6 (*n*=5). (**B**) The protein level of SCF in gastric smooth muscles was obviously reduced from DM week 6 (*n*=5; *P<0.05).

**Figure 5:**
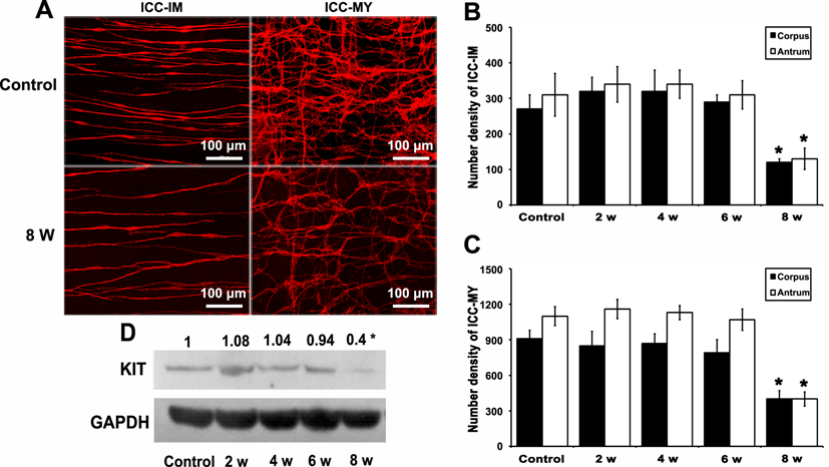
Decreased ICCs and KIT expression in DM week 8. (**A**) In the whole mount preparations, ICC-IM were fusiform bipolar cells; and ICC-MY had 2–4 slender processes which ramified to form cellular networks. In DM week 8, ICC-IM were obviously lost when compared with the controls. Cellular networks of ICC-MY were much more sparse in DM week 8 (*n*=5). (**B**) The number density of ICC-IM in gastric corpus and antrum of DM mice were decreased in DM week 8 compared with the controls (*n*=5; *P<0.05). (**C**) The number density of ICC-MY in gastric corpus and antrum of DM mice were decreased in DM week 8 compared with the controls (*n*=5; *P<0.05). (**D**) The protein level of KIT was not significantly decreased until DM week 8 (*n*=5; *P<0.05).

SCF is mainly synthesized and secreted by the smooth muscles and myenteric neurones in the gastrointestinal tract. Here, we showed that SCF was present in the gastric smooth muscles and myenteric neurones but not in ICC (Figure 4A), consistent with the distribution of IGF-1R and InsR (Supplementary Figure S3). The gastric SCF was clearly decreased from DM week 6 (*P<0.05, Figure 4A,B). Whereafter, in DM week 8, the cellular networks of ICC were deteriorated (Figure 5A) and the number densities of ICC-IM and ICC-MY were markedly decreased (Figure 5B,C), parallel with the attenuated KIT (*P<0.05, Figure 5D). These results suggested that the impaired IGF-1/IGF-1R and insulin/InsR systems damaged ICC by inhibiting SCF production instead of directly affecting ICC.

### Insulin delayed the development of diabetic gastroparesis

**Figure 6:**
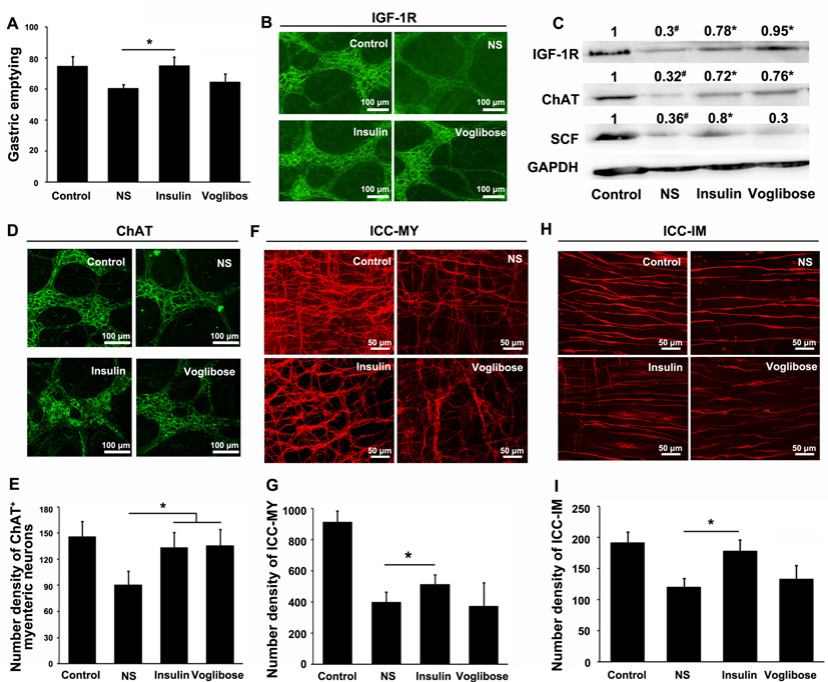
Effects of 8-week insulin or Voglibose treatment (**A**) The gastric emptying of the DM mice treated with insulin was accelerated compared with that of the DM mice receiving NS (*n*=5; *P<0.05); while the gastric emptying of the DM mice treated with Voglibose was not significantly improved. (**B**) The immunoreactive intensity for IGF-1R in the myenteric plexuses of the insulin and Voglibose groups was obviously increased compared with the NS group (*n*=10). (**C**) Compared with the controls, the protein levels of gastric IGF-1R, ChAT, and SCF were significantly decreased in the DM mice that only received NS for 8 weeks (*n*=5; ^#^*P*<0.05). The DM-associated decrease in IGF-1R and ChAT were clearly restored by insulin or Voglibose compared with the NS group (*n*=5; *P< 0.05). However, only insulin had positive effect on the SCF production (*n*=5; *P<0.05 compared with NS). (**D**) The immunoreactive intensity for ChAT in the myenteric plexus was increased after insulin or Voglibose treatment (*n*=10). (**E**) The number density of ChAT^+^ myenteric neurones was well protected upon the treatment of insulin or Voglibose compared with the NS group (*n*=10; *P<0.05). (**F**–**I**) Insulin partly recovered the count and cellular networks of ICC-MY and ICC-IM that were labeled with KIT; while Voglibose had no obvious effect on ICC (*n*=10; *P<0.05).

It was recently proposed that the early usage of insulin could delay DM complications. Therefore, we treated the DM mice with insulin or Voglibose for 8 weeks right after DM was induced. The delayed gastric emptying of the DM mice was efficiently improved by the 8-week insulin administration (24.0%, *P<0.05) but not Voglibose compared with the DM mice receiving NS (Figure 6A). The gastric IGF-1R in the insulin and Voglibose groups were evidently increased compared with the NS group (*P<0.05; Figure 6B,C). The gastric ChAT was not decreased and the number of ChAT^+^ myenteric neurones was well preserved by the treatment of insulin or Voglibose (*P<0.05; Figure 6C–E). Notably, insulin not Voglibose treatment was able to increase SCF production in the gastric smooth muscles (*P<0.05; Figure 6C). Moreover, only insulin treatment could partly prevent the loss of ICC-MY (28.8%, *P<0.05) and ICC-IM (48.3%, *P<0.05) and their cellular networks (Figure 6F–I).

## Discussion

In the present study, type 1 DM mouse model was successfully made by an injection of Alloxan monohydrate, which resulted in apparent declines in the serum insulin by 62.2–78.9% and IGF-1 by 12.1–43.8% from as early as DM week 2. Four weeks after the onset of the DM, the gastric emptying was delayed indicating a link between the reduced insulin/IGF-1 and diabetic gastroparesis. The amplitude of gastric muscle contraction was decreased but the frequency of the gastric muscle contraction was increased in DM week 4, which may be due to compensation for the gastric movement. Then, the amplitude and the frequency were reduced compared with controls.

The ENS plays a crucial role in regulating gastrointestinal movement. The number and morphometric changes in the enteric neurones attributed to increased apoptosis, oxidative stress, and hyperglycemia likely participated in the development of diabetic gastroparesis in mice, rats, and humans [18–20]. In the current study, the myenteric neuropathy might arise as a consequence of reduced insulin/InsR and IGF-1/IGF-1R in DM mice because they are considered as neurotrophic factors. Myenteric neuronal population comprises excitatory neurones, inhibitory neurones, and interneurones. Previous studies have revealed an abnormality of inhibitory neuronal nitric oxide synthase (nNOS) neurones in diabetic gastroparesis [21,22]. Here, we had a special interest in excitatory enteric neurones that keep gastric motility balance in co-operation with inhibitory neurones. Our results showed that the InsR^+^/IGF-1R^+^ neurones were mainly ChAT^+^ excitatory cholinergic neurones. In view of that, the insulin/InsR and IGF-1/IGF-1R signaling play a key role in the neuronal survival and development, the decreased insulin/InsR and IGF-1/IGF-1R might not fully maintain the ChAT^+^ cholinergic neurones viable. Consequently, the gastric ChAT and the number density of ChAT^+^ myenteric cholinergic neurones were diminished from DM week 4, which aggravated the diabetic gastroparesis progress.

Apart from the ENS, ICC also play a key role in the gastrointestinal motility as the pacemaker of spontaneous slow waves within the gastrointestinal tract and the mediator of neuronal transmission from the ENS to gastrointestinal smooth muscles. ICC are mainly distributed in the tunica muscularis (ICC-IM) and myenteric plexus (ICC-MY) within the gastric wall. ICC-IM are spindle-shaped bipolar cells, and ICC-MY have 2–4 slender processes which ramify to form cellular networks. The depletion of ICC in the gastrointestinal tract is responsible for the diabetic gastroparesis both in type 1 and type 2 DM patients and mice [22–24]; and the loss of ICC in diabetic gastroparesis was related with the deficiency of IGF-1 and/or insulin [25]. The progenitors of ICC (Kit^low^CD44^+^CD34^+^InsR/IGF-1R^+^) could not develop mature cells (Kit^+^CD44^+^CD34^−^InsR/IGF-1R^−^) in the absence of insulin and IGF-1; and the survival and function of ICC would not be well preserved either [6,26]. However, we failed to detect IGF-1R and InsR in ICC [27]. These results raised a question: how IGF-1/insulin affects ICC? It is well known that the growth, survival, and function of ICC are largely dependent on the activation of the membrane receptor KIT by its natural ligand SCF [28,29]. Therefore, SCF deficiency may lead to the loss of ICC via impairing the KIT/SCF signaling pathway. As expected, in our DM mice, following the reduced insulin/InsR and IGF-1/IGF-1R signaling, the SCF production by the gastric smooth muscles was clearly decreased in DM week 6, and ICC were significantly lost afterward (in DM week 8). The above evidence inferred that the protective effect of insulin and IGF-1 on ICC might be via indirect way of facilitating SCF production. This hypothesis was supported by the fact that smooth muscles coexpressed InsR and IGF-1R as well as SCF, and the SCF expression in the gastric smooth muscles was restrained by IGF-1R inhibitor [30].

Early usage of insulin has been favored in recent years to treat DM and its complications. Here, we treated the DM mice with either insulin or Voglibose for 8 weeks. The gastric emptying was efficiently improved by insulin treatment, while glycemic control by Voglibose failed in accelerating the gastric emptying, indicating that simple glycemic control was unable to efficiently delay diabetic gastroparesis developing. A clinical trial by Russo et al. [14] has consolidated the contribution of insulin to accelerate gastric emptying in long-standing type 1 DM; and they thought it was owing to the insulin-induced hypoglycemia. In the present study, we suggested that the protective role of insulin/InsR and IGF-1/IGF-1R signaling on the myenteric cholinergic neurones and ICC was also contributory. Insulin treatment increased InsR and IGF-1R expressions in the myenteric plexuses and prevented the loss of myenteric cholinergic neurones. It was reported that insulin and IGF-1 increased ChAT activity in brain tissue and cultured human central nervous system derived neuronal cells [31]. In addition to the findings in the central nervous system, our results demonstrated that insulin was beneficial for the survival of the myenteric ChAT^+^ neurones. Besides, insulin was helpful for the maintenance of ICC networks by stimulating SCF production. We also found that InsR and IGF-1R were increased upon Voglibose treatment, so were the ChAT expression and myenteric cholinergic neurones. A recent study revealed that Acarbose, an analogue of Voglibose, enhanced the level of hippocampal InsR in old mice [32]. We presumed here that hyperglycemia might abate InsR and IGF-1R expressions in the myenteric ChAT^+^ neurones but the detailed mechanism needs to be further clarified. However, Voglibose was insufficient to increase SCF production and hardly exhibited protection on ICC. There seemed to be a contradiction that Voglibose-induced InsR and IGF-1R expression in the gastric wall did not promote SCF production. It might because insulin/InsR and IGF-1/IGF-1R signaling are not the only factors that influence SCF production. For example, ghrelin was reported to be capable of enhancing SCF expression in rats’ stomachs [33]. ICC gained a central place in the gastrointestinal motility because they integrate excitatory and inhibitory neurotransmission with intestinal slow-wave activity [34]. Once ICC are dysfunctional, the neurotransmission of myenteric neurones will be retarded, which partly interprets that simple glycemic control by Voglibose was unable to delay diabetic gastroparesis developing even if it well protected myenteric ChAT^+^ neurones. For this reason, we believe that prophylactic insulin in DM patients not only because it restores normoglycemia, but also preserves ICC.

In conclusion, we demonstrated that the myenteric ChAT^+^ excitatory neurones were lost with the decreased insulin/InsR and IGF-1/IGF-1R signaling in the earlier stage of DM. Along with the further decrease in IGF-1/IGF-1R and insulin/InsR, SCF production by the gastric smooth muscles was clearly repressed, resulting in loss of ICC and impairment of their cellular networks in the latest DM stage. Significantly, compared with Voglibose, insulin treatment effectively delayed diabetic gastroparesis from developing probably via retrieving insulin/InsR and IGF-1/IGF-1R signaling that recovered the ChAT^+^ excitatory neurones and ICC. Therefore, we suggested prophylactic insulin therapy may help to delay or even prevent the diabetic gastroparesis.

## Supporting information

**Table S1:** Number of mice used in each group

**Table S2:** Antibodies

**Table S3:** Blood glucose and body weight

**Table S4:** Serum Insulin and IGF-1

## Abbreviations

AOI: area of interest
ChAT: choline acetyltransferase
DM: diabetes mellitus
ENS: enteric nervous system
HRP: horseradish peroxidase
ICC: interstitial cell of Cajal
IGF-1: insulin-like growth factor-1
NS: normal saline
PFA: paraformaldehyde
SCF: stem cell factor
