# Supplementary figures and images for: Impaired insulin/IGF-1 is responsible for diabetic gastroparesis by damaging myenteric cholinergic neurones and interstitial cells of Cajal
Source: Biosci Rep. 2017 Oct 27;37(5):BSR20170776. doi: 10.1042/BSR20170776 (PMC5665615; doi:10.1042/BSR20170776)

**Control**

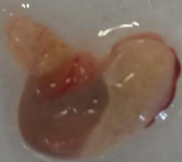

1 cm

**8 W**

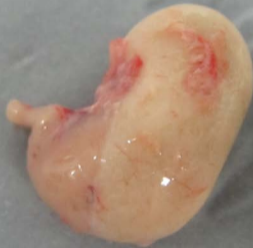

1 cm

**Control**

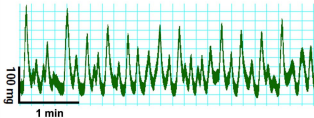

**4 w**

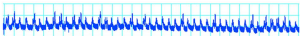

**6 w**

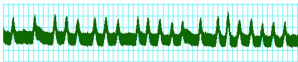

**8 w**

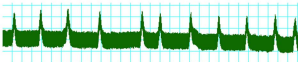

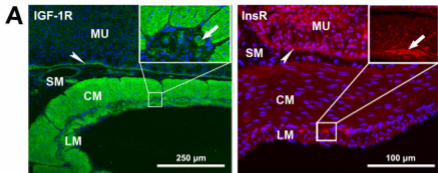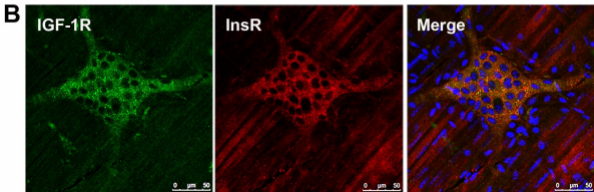

**A****IGF-1R**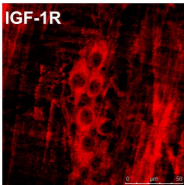**ChAT**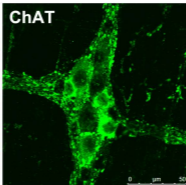**Merge**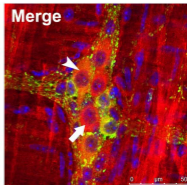**B****InsR**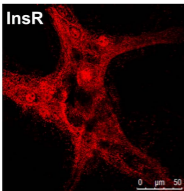**ChAT**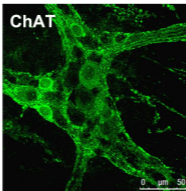**Merge**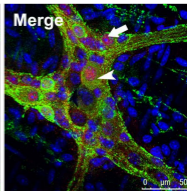

**A**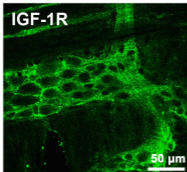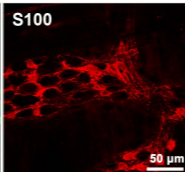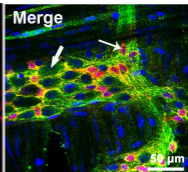**B**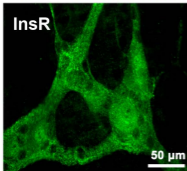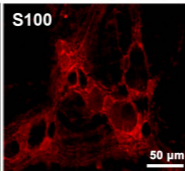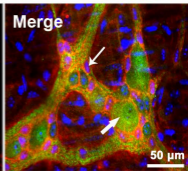

Supplement: Supplementary file 1 [file BSR20170776_Supp1.pdf]
