# Supplementary material for: Impaired insulin/IGF-1 is responsible for diabetic gastroparesis by damaging myenteric cholinergic neurones and interstitial cells of Cajal
Source: Biosci Rep. 2017 Oct 27;37(5):BSR20170776. doi: 10.1042/BSR20170776 (PMC5665615; doi:10.1042/BSR20170776)
Supplement: Supplementary file 2 [file BSR20170776_Supp2.pdf]

Table S1 Number of mice used in each group

Group	Total number	Gastric emptying	Western blot	Cryosection	Whole mount preparation (in 4% paraformaldehyde)	Whole mount preparation (in 100% acetone)
Control	15		5		5	5
DM 2 w	15		5		5	5
DM 4 w	15		5		5	5
DM 6 w	15		5		5	5
DM 8 w	15		5		5	5
DM + NS 8 w	15		5		5	5
DM + Insulin 8 w	30	5	5	-	10	10
DM + Voglibose 8 w	30	5	5	-	10	10

Table S2 Antibodies

Antibody	Supplier	Western blot	Immunofluorescensce staining
rabbit anti-IGF-1R	Santa Cruz, USA	1:1000	1:100
goat anti-IGF-1R	Santa Cruz, USA		1:100
rabbit an ti-InsR	Abcam, USA	1:1000	1:100
goat anti-InsR	Santa Cruz, USA		1:100
goat anti-ChAT	Novus biologicals, USA	1:800	1:100
rabbit anti-S100	Boster, Wuhan, China		1:100
mouse anti-SCF	Santa Cruz, USA	1:1000	1:100
rat anti-KIT	eBioscience, USA		1:200
rabbit anti-KIT	Santa Cruz, USA	1:400	
goat anti-GAPDH	Santa Cruz, USA	1:4000	
HRP-conjugated donkey anti-goat IgG	Santa Cruz, USA	1:4000	
HRP-conjugated goat anti-mouse IgG	Santa Cruz, USA	1:4000	
HRP-conjugated goat anti-rabbit IgG	Santa Cruz, USA	1:2000	
FITC-conjugated goat anti-rat IgG	Life Technologies, USA		1:400
FITC-conjugated goat anti-rabbit IgG	Life Technologies, USA		1:400
Cy3-conjugated goat anti-rabbit IgG	Life Technologies, USA		1:400
Cy3-conjugated goat anti-mouse IgG	Life Technologies, USA		1:400
Alexa Fluor 488-conjugated donkey anti-goat IgG	Life Technologies, USA		1:400
Alexa Fluor 594-conjugated donkey anti-goat IgG	Life Technologies, USA		1:400
Cy3-conjugated goat anti-rat IgG	Life Technologies, USA		1:400

Table S3 Blood glucose and body weight

Group (n = 15)	Glucose (mmol/L)	Body weight (g)
Control	4.5 ± 0.5	22.7 ± 0.9
DM 2 w	25.1 ± 4.8*	19.1 ± 2.1*
DM 4 w	31.4 ± 2.6*	18.6 ± 2.2*
DM 6 w	31.3 ± 2.3*	18.6 ± 1.8*
DM 8 w	30.5 ± 2.7*	17.6 ± 2.2*

* *P* < 0.05 compared with Control

Table S4 Serum Insulin and IGF-1

Group (n = 6)	Insulin (ng/mL)	IGF-1 (ng/mL)
Control	0.90 ± 0.18	154.88 ± 22.36
DM 2 w	0.34 ± 0.09*	136.21 ± 15.91*
DM 4 w	0.33 ± 0.09*	100.22 ± 15.62*
DM 6 w	0.29 ± 0.08*	95.80 ± 10.22*
DM 8 w	0.19 ± 0.06*	87.11 ± 12.54*

* *P* < 0.05 compared with Control
